# Measurement of the temporal latency of a respiratory gating system using two distinct approaches

**DOI:** 10.1002/acm2.13768

**Published:** 2022-09-09

**Authors:** Michael G. Stock, Connel Chu, Jonas D. Fontenot

**Affiliations:** ^1^ Department of Radiation Oncology Thomas Jefferson University Hospital Philadelphia PA USA; ^2^ Department of Physics Mary Bird Perkins Cancer Center Baton Rouge LA USA

**Keywords:** gating system, latency, motion management, response

## Abstract

**Purpose:**

To develop a methodology that can be used to measure the temporal latency of a respiratory gating system.

**Methods:**

The gating system was composed of an automatic gating interface (Response) and an in‐house respiratory motion monitoring system featuring an optically tracked surface marker. Two approaches were used to measure gating latencies. A modular approach involved measuring separately the latency of the gating system's complementary metal–oxide–semiconductor tracking camera, tracking software, and a gating latency of the LINAC. Additionally, an end‐to‐end approach was used to measure the total latency of the gating system. End‐to‐end latencies were measured using the displacement of a radiographic target moving at known velocities during the gating process.

**Results:**

Summing together the latencies of each of the modular components investigated yielded a total beam‐on latency of 1.55 s and a total beam‐off latency of 0.49 s. End‐to‐end beam‐on and beam‐off latency was found to be 1.49 and 0.34 s, respectively. In each case, no statistically significant differences were found between the end‐to‐end latency of the gating system and the summation of the individually measured components.

**Conclusion:**

Two distinct approaches to quantify gating latencies were presented. Measuring the end‐to‐end latency of the gating system provided an independent means of validating the modular approach. It is expected that the beam‐on latencies reported in this work could be reduced by altering the control system configuration of the LINAC. The modular approach can be used to decouple the individual latencies of the gating system, but future improvements in the temporal resolution of the service graphing feature are needed to reduce the uncertainty of LINAC‐related gating latencies measured using this approach. Both approaches are generalizable and can be used together when designing a quality assurance program for a respiratory gating system.

## 1 INTRODUCTION

Respiratory gating has been used in radiation therapy to decrease intrafraction motion by limiting treatment delivery to particular phases or amplitudes of a patient's respiratory cycle.^1^ In some cases, respiratory gating can be used to decrease the internal margins of the planning target volume, allowing for dose escalation without increased normal tissue complications.^2^, ^3^ A successful implementation of this technique requires systems to accurately track the patient's respiratory cycle via internal or external surrogates. When the surrogate used for respiratory tracking is within the predefined phase or amplitude range, referred to as the gate window, the system must be able to trigger on the radiation beam automatically. Gating latency refers to the time delay between the respiratory surrogate entering or leaving the gate window and when radiation delivery begins or ends, respectively. Minimizing these time delays is critical for systems used for respiratory gating because failure to gate the beam at the appropriate time can decrease the efficiency of treatment delivery or could lead to a geometric miss of the target.^4^

End‐to‐end latency refers to the total time delay of the entire gating system. Several authors have presented methods for measuring gating latencies using the displacement of a radio‐opaque moving target captured using film or the LINACs electronic portal imaging device (EPID).^4^, ^5^, ^6^, ^7^ Such methods can be used to measure the end‐to‐end latencies of the gating system but do not allow for analysis of the individual components included in the system. Modern gating systems are typically composed of multiple components, or subsystems, each having their own latency. Saito et al.^8^ presented a method of measuring the individual component latencies of their gating system directly using a multichannel oscilloscope. To the best of the author's knowledge, no study has measured both end‐to‐end and component latencies of a gating system separately to compare to one another.

This work presents two distinct approaches to measure the latency of a respiratory gating system. One of these approaches can be used to measure the latencies of the gating system's individual components, whereas the other can be used to measure the gating system's total end‐to‐end latency.

## 2 METHODS

**FIGURE 1:**
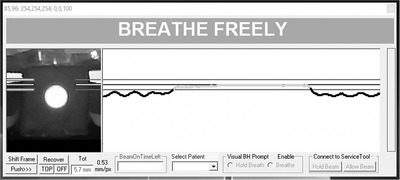
In‐house tracking software showing the complementary metal–oxide–semiconductor (CMOS) camera's streamed view of the reflective marker (left), respiratory trace based on the marker's centroid position, and gate window with user‐configurable gating thresholds

**FIGURE 2:**
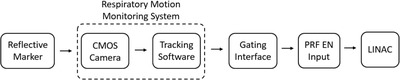
Schematic diagram of the respiratory gating system. A reflective marker was optically tracked using an in‐house respiratory motion monitoring system. When the marker's displacement exceeds user‐configurable gating thresholds, the gating interface would signal to the LINAC to pause treatment delivery via PRF EN inputs.

This work utilized a commercially available gating interface (Response, Elekta AB, Stockholm, Sweden) to connect a respiratory motion monitoring system to a LINAC (Elekta Versa HD, Elekta AB, Stockholm, Sweden). The respiratory motion monitoring system was created at our institution and was designed to track the 1‐D vertical displacement of a reflective marker placed on the patient's abdominal surface. The marker was optically tracked using a complementary metal–oxide–semiconductor (CMOS) 2‐Megapixel camera with a USB 2.0 interface and CMOS OV2710 sensor (ELP, Model:ELP‐USBFHD05MT‐KL36IR). The camera was positioned on the treatment couch 80 cm from the marker. Both were aligned on the treatment couch using the room's wall‐mounted sagittal wall laser to provide a reproducible setup. A computer with an Intel Core i5 processor (EliteBook, HP, Palo Alto, CA) was used to run the in‐house Windows‐based software that would track the centroid position of the reflective marker (Figure 1). This computer was connected to the gating interface via USB connection. When marker displacement exceeded user‐configurable thresholds, the gating interface would signal the LINAC control system to pause treatment delivery via the pulse repetition frequency enable (PRF EN) input.^9^ A schematic diagram of the respiratory gating system studied in this work is shown in Figure 2.

### 2.1 Modular approach

A modular approach was used to characterize the latencies of the respiratory gating system. With this approach, the latencies of the individual components of the gating system were evaluated separately, which included the CMOS camera, the tracking software, and the gating latencies of the LINAC. The CMOS camera was used to optically track a reflective marker. Therefore, streaming latency present in the camera and display system increases the time it takes for marker displacements to be detected by the gating interface.

**FIGURE 3:**
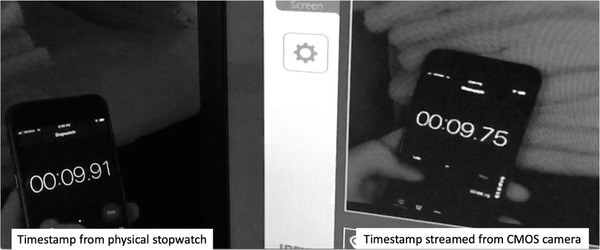
A physical stopwatch and streamed view of the stopwatch were captured in the same image. The streaming latency of the complementary metal–oxide–semiconductor (CMOS) camera was calculated as the difference between the timestamp shown on the physical stopwatch and the timestamp that was streamed from the camera.

The streaming latency of the CMOS camera was measured by recording a running stopwatch. The camera was connected to a monitor, which displayed a streamed view of this stopwatch. A separate camera was used to capture the physical stopwatch and the streamed view of the stopwatch in the same image (Figure 3). The difference between the timestamp shown on the physical stopwatch and timestamp that was streamed from the CMOS camera was used to measure the camera's streaming latency.

The sampling rate of the tracking software represents the frequency at which the software could update the position of the reflective marker. The tracking software was designed to produce log files listing the marker's positions and associated timestamps during use of the software. The sampling rate of the tracking software was confirmed using successive timestamps shown in these log files.

With the modular approach, the gating latency of the LINAC represented the time delay between the LINAC control system receiving input from the gating interface and radiation delivery beginning or ending. LINAC gating latencies were measured using the graphing feature of the LINAC's control system. The graphing feature was accessed in service mode and can be used to plot various treatment control system variables with respect to time on the same set of axes. Item 44 part 4 was used to measure the dose rate of the LINAC, and item 2201 part 190 was used to measure the PRF EN input. The PRF EN input was used to determine the time at which the gating interface signaled the LINAC control system to pause or resume treatment delivery. These variables were plotted simultaneously as a function of time to measure the beam‐on and beam‐off gating latencies of the LINAC. There is some time that it takes for the LINAC to ramp up and settle into the programmed dose rate during beam delivery. For consistency and reproducibility, we chose to define LINAC beam‐on latency as the time between the LINAC control system receiving the PRF EN input and the dose rate increasing to 80% of its maximum value. Alternatively, the LINAC beam‐off latency was defined as the time between the gating interface interrupting the PRF EN input and the dose rate decreasing to 20% of its maximum value. A 6‐MV beam energy was used for all measurements in this study. Ten separate gating cycles were used to measure average LINAC gating latencies.

### 2.2 End‐to‐end approach

An end‐to‐end approach was used to measure the total beam‐on and beam‐off latencies of the respiratory gating system. Gated images of a radiographic target were acquired at various velocities to calculate total system latencies. A linear relationship is expected between the displacement of the radiographic target from a reference position and its velocity during the gating process. Therefore, end‐to‐end latency of the gating system was computed as the slope the target's displacement plotted as a function of velocity.

**FIGURE 4:**
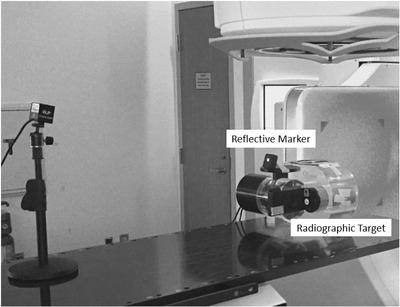
A respiratory motion phantom was used to measure end‐to‐end latencies of the gating system. The motion of an optically tracked reflective marker and radiographic target were synchronized so that gated images of the target could be captured with the electronic portal imaging device (EPID) when the reflective marker entered the gate window. The reflective marker was able to move in the vertical direction, and the radiographic target was set to move only in the longitudinal direction.

A commercially available respiratory motion phantom (QUASAR Respiratory Phantom, Modus Medical Devices, Ontario, Canada) was used to synchronize the motion of an optically tracked reflective marker and a radiographic target. This allowed gated images of the radiographic target to be captured when the marker entered the gate window. The radiographic target was a 3‐cm diameter plastic sphere embedded in a cylindrical cedar insert. The radiographic target was set to move only in the longitudinal direction with displacements ranging from ±1.5 cm. The reflective marker was secured to the chest wall platform of the phantom and was monitored using the gating system's CMOS camera (Figure 4). The EPID (iViewGT, Elekta AB, Stockholm, Sweden) was used to capture images of the radiographic target using single exposure and maximum frame averaging settings.

The reference position used for the gated images corresponded to one of the user‐configurable gating thresholds. The gating thresholds were chosen because they trigger the beam on or off during gated deliveries. Therefore, imaging the target at this position shows its location under the ideal conditions of no latency present in the system. To set the target at the reference position, the amplitude of the chest wall platform of the phantom was manually adjusted while monitoring the respiratory trace in the tracking software. Once the respiratory trace reached the lower amplitude gating threshold, the tracking software would alert the user, and this phantom position would be used to acquire the reference image. Therefore, the accuracy that the reflective marker could be set to the reference position was related to the pixel size used within the tracking software, which was on the order of 1 mm. An MV portal image was acquired of the static target at the reference position using 100‐MU and a 6‐MV beam energy.

Gated images of the target traveling at constant velocity were acquired with the phantom and gating thresholds kept in the same positions that were used to collect the reference image. This ensured that displacement of the target from the reference position in each image would be the result of the end‐to‐end latencies present in the gating system. The phantom was programed with custom respiratory traces of ramp functions so that the radiographic target would be moving at constant velocity while the gated images were acquired. To measure beam‐on latency, the respiratory phantom was programed to move the target into the gate window. For beam‐off latency, the phantom was programed to begin inside of the gate window and while the beam was being delivered the target was driven out the gate window with constant velocity. The target velocities used in this study ranged from 1 mm per second to 48 mm per second. Images of the moving target were captured with the EPID and were averaged together to produce a single image of the target's travel while the beam was on.

**FIGURE 5:**
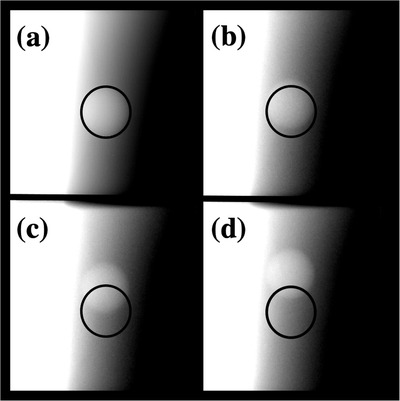
Images of a radiographic target moving at constant velocity were used to measure end‐to‐end latency of the gating system. The reference position was contoured and overlaid on each of the images. Images used to measure beam‐on latency are shown for target velocities of (a) 0 mm per second (reference position), (b) 1.5 mm per second, (c) 4.5 mm per second, and (d) 7.5 mm per second.

**FIGURE 6:**
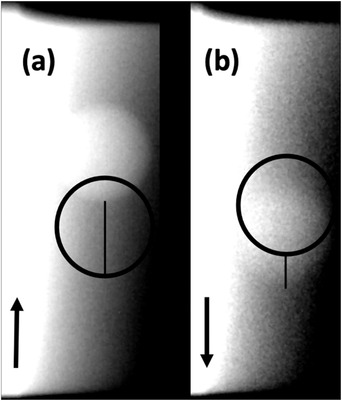
The displacement from the reference position was measured on each gated image. The direction of target travel is specified by the arrow in each image: (a) displacement due to beam‐on latency with the target moving at 7.5 mm per second; (b) displacement due to beam‐off latency with the target moving at 32 mm per second.

All image analysis was performed using open‐source image processing software (ImageJ, National Institute of Health).^10^ No filters were applied, and all images were window and leveled with the same settings to improve target contrast and obtain consistent image quality. The target was manually contoured on the reference image, and the contour was overlaid on all of the other images in the set (Figure 5). The known diameter of the target was used to calibrate the image processing software so that absolute distances could be measured within each image. The distance from the reference position contour to the same corresponding point on the target was manually measured on each image. The direction that the target was traveling when the images were acquired was taken into account. For instance, the target's travel during beam delivery was included when measuring beam‐off latency and excluded when measuring beam‐on latency (Figure 6).

**FIGURE 7:**
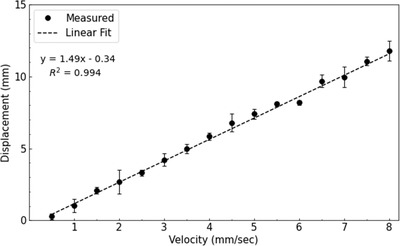
Target displacements resulting from beam‐on latency. Target velocities of 0.5–8.0 mm per second were used. The error bars represent the standard deviation of repeat measurements taken at each target velocity. The slope of a linear fit was used to calculate the end‐to‐end beam‐on latency of 1.49 s.

**FIGURE 8:**
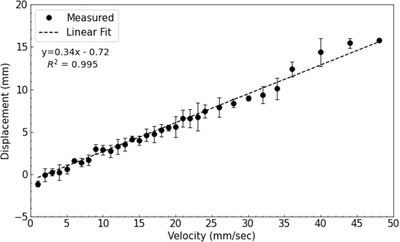
Target displacements resulting from beam‐off latency. Target velocities of 1–48 mm per second were used. The error bars represent the standard deviation of repeat measurements taken at each target velocity. The slope of a linear fit was used to calculate the end‐to‐end beam‐off latency of 0.34 s.

Average displacements were calculated from the set of repeat measurements collected at each target velocity. Target displacements due to beam‐on and beam‐off latencies were plotted separately against the known target velocities (Figures 7 and 8). The error bars shown in each figure represent the standard deviation of the repeat measurements performed at each target velocity. A weighted least squares fit was applied to the data, and the slope of the best fit line was used to quantify the end‐to‐end latency of the gating system. To compare the results of the modular and end‐to‐end approaches, a two‐tailed independent *t*‐test was used to test for significant differences between them.

## 3 RESULTS

The measured latencies for each of the components investigated using the modular approach are listed in Table 1. The sum of these modular latencies yielded total a beam‐on time delay of 1.55 ± 0.25 s and a total beam‐off delay of 0.49 ± 0.25 s. Based on the data from the component measurements, the LINAC gating latency was the largest contributor to both beam‐on and beam‐off time delays. The streaming latency of the tracking camera was 150 ms and had a relatively large impact on the gating system's total beam‐off latency.

**TABLE 1:** Latencies of the respiratory gating system measured using the modular approach

Gating system component	Latency (s)
CMOS tracking camera	0.15 ± 0.02
Tracking software (time between samples)	0.052 ± 0.011
LINAC beam‐on latency	1.35 ± 0.25
LINAC beam‐off latency	0.29 ± 0.25
Total beam‐on latency	1.55 ± 0.25
Total beam‐off latency	0.49 ± 0.25

Abbreviation: CMOS, complementary metal–oxide–semiconductor.

The results of the end‐to‐end approach for beam‐on and beam‐off latencies are shown in Figures 7 and 8, respectively. Target velocities used to measure beam‐on latency were limited to 8 mm per second because larger velocities caused the respiratory motion phantom to reach its maximum displacement before beam delivery could begin. Based on the slope of the linear fit applied to each dataset, the end‐to‐end beam‐on and beam‐off latencies were found to be 1.49 ± 0.03 and 0.34 ± 0.01 s, respectively. No significant difference in beam‐on latency was observed (*t*(14) = 0.21, *p* = 0.83) between the modular and end‐to‐end approaches. Similarly, no significant difference in beam‐off latency was observed (*t*(31) = 0.56, *p* = 0.58) between the two approaches presented.

## 4 DISCUSSION

Negative offset values were observed for each of the linear fits shown in Figures 7 and 8. Ideally each offset should be zero, because at zero velocity, there should be no displacement of the target from the reference position. This suggests a systematic error in our experimental methodology, which could have resulted from incorrectly positioning the reflective marker at the gating threshold when establishing the reference position. However, this is not expected to impact the latencies reported in this work because the resulting offsets would not affect the slope of the linear fits performed. Woods and Rong^6^ performed similar measurements and also observed negative offset values, which they discussed was likely the result of measurement uncertainty and finite pixel size. In our work, the observed offsets were within the pixel size used to track the reflective marker.

Target displacements shown in Figure 8 were found to deviate from the linear fit at high target velocities. This is likely the result of the target passing the gating threshold before it accurately reached its programmed velocity. To assess the impact that these data points had on the reported beam‐off latency, the linear fit was also performed without data from target velocities greater than 24 mm per second. Excluding these data points changed the resultant latency by less than 30 ms. Therefore, the inclusion of these high velocity measurements did not impact the major findings reported in this work.

One of the limitations of the end‐to‐end approach was that target displacement was manually measured on each gated image, which is susceptible to inconsistencies in defining the edge of the target due to motion blurring at high target velocities. Multiple measurements were made at each target velocity to improve the accuracy of this method. An improvement to this technique could involve multiple people measuring the target displacement in each image or automated measurements to improve consistency. Additionally, utilizing a target with high radiographic contrast may improve the ability to delineate the edge of the target.

Others in the literature have reported considerably smaller beam‐on latencies than those reported in this work using the modular or end‐to‐end approaches.^5^, ^8^ Within the control system of the LINAC, several parameters can be adjusted to alter the temporal characteristics of gated delivery, particularly with regards to beam‐on latency.^9^, ^11^ It is expected that the beam‐on latencies reported in this work could be reduced by altering these parameters. However, there is some tradeoff between the time it takes for the LINAC to begin producing radiation pulses and the dosimetric stability of those pulses during beam startup. A potential consequence of altering the control system configuration of the LINAC to diminish the gating system's beam‐on latency is dosimetric degradation, which was not studied in this particular work. At our institution, this beam‐on latency was clinically acceptable given that the gating system is intended for use with the deep inspiration breath‐hold technique. However, for systems that are intended for free‐breathe gating applications, optimizing the control system configuration of the LINAC to minimize the gating system's beam‐on latency may be necessary.

One of the benefits of utilizing a modular approach when characterizing gating latency is that it allows the contribution of the system's individual components to be decoupled from the end‐to‐end total. For instance, we found that the latencies of this gating system were dominated by the beam‐on and beam‐off time delay of the LINAC. The streaming latency of the tracking camera also had a considerable impact, especially for beam‐off latency given its relatively small magnitude. This type of analysis can provide insight into which components of the gating system should be targeted when trying to reduce system latencies.

The modular approach can be generalized to other gating systems that utilize different components than those studied in this work. Currently, there are several commercially available systems that employ optical tracking. The streaming latency of the cameras utilized in these other systems could be measured with the same technique used in this work. For gating systems that employ other forms of respiratory motion monitoring the user could either measure their latency directly, or, use published data for their specific system. The LINAC gating latency of the users system could be measured using the graphing feature of the LINAC control system. This novel method provides a convenient way to investigate the impact that changes to the control system configuration of the LINAC has on gating latency. However, one of the limitations of this method is that the 4‐Hz sampling frequency of the service graphing feature limits the precision at which LINAC gating latencies can be measured.

The end‐to‐end approach can also be generalized to other gating systems as long as the motion of a respiratory phantom can be coupled to the gating system's respiratory motion monitoring system. This approach was able to measure system latencies with less uncertainty than the modular approach and could be used as a method of independent validation. Disagreement between the results of the two approaches could demonstrate that not all of the gating system's components were included in the modular analysis.

Routine quality assurance (QA) for respiratory gating systems is recommended by the report of Task Group 142 of the American Association of Physicists in Medicine.^12^ Gating latency should be measured periodically and compared against baseline values established during commissioning of the system. The approaches presented in this work to measure gating latency could be used when designing a QA program for the gating system. During commissioning, baseline latency values could be measured using both approaches. This allows for flexibility when performing routine QA of the system. For instance, the modular approach could be used on a more frequent basis as a spot check or following changes to any of the components of the gating system. The end‐to‐end approach can also be used to perform the recommended QA of the gating system, especially when less uncertainty is needed.

## 5 CONCLUSION

In this work, two distinct approaches to quantify gating latencies were presented. End‐to‐end measurements provided an independent means of validating the total system latencies found using the modular approach. The modular approach can be used to decouple the individual latencies of the gating system, but future improvements in the temporal resolution of the service graphing feature are needed to reduce the uncertainty associated with latencies measured using this approach. Both approaches can be generalized to other respiratory gating systems and can be used together when designing a QA program.

## CONFLICT OF INTEREST

The authors have no conflicts of interest to disclose.

## AUTHOR CONTRIBUTION

Michael G. Stock collected the data and drafted the manuscript. Connel Chu was responsible for creating the gating system studied in this particular work and providing insight on possible ways to measure gating latencies of this novel system. Jonas D. Fontenot was responsible for providing expertise on the direction and scope of the project, reviewing the data, and editing and reviewing the manuscript.

## Data Availability

The data that support the findings of this study are available from the corresponding author upon reasonable request.
